# Nucleus Tractus Solitarius Neurons Activated by Hypercapnia and Hypoxia Lack Mu Opioid Receptor Expression

**DOI:** 10.3389/fnmol.2022.932189

**Published:** 2022-07-11

**Authors:** Sebastian N. Maletz, Brandon T. Reid, Adrienn G. Varga, Erica S. Levitt

**Affiliations:** ^1^Department of Pharmacology and Therapeutics, University of Florida, Gainesville, FL, United States; ^2^Breathing Research and Therapeutics Center, University of Florida, Gainesville, FL, United States

**Keywords:** opioid, nucleus of the solitary tract, respiratory depression, hypercapnia, hypoxia

## Abstract

Impaired chemoreflex responses are a central feature of opioid-induced respiratory depression, however, the mechanism through which mu opioid receptor agonists lead to diminished chemoreflexes is not fully understood. One brainstem structure involved in opioid-induced impairment of chemoreflexes is the nucleus of the solitary tract (NTS), which contains a population of neurons that express mu opioid receptors. Here, we tested whether caudal NTS neurons activated during the chemoreflex challenge express mu opioid receptors and overlap with neurons activated by opioids. Using genetic labeling of mu opioid receptor-expressing neurons and cFos immunohistochemistry as a proxy for neuronal activation, we examined the distribution of activated NTS neurons following hypercapnia, hypoxia, and morphine administration. The main finding was that hypoxia and hypercapnia primarily activated NTS neurons that did not express mu opioid receptors. Furthermore, concurrent administration of morphine with hypercapnia induced cFos expression in non-overlapping populations of neurons. Together these results suggest an indirect effect of opioids within the NTS, which could be mediated through mu opioid receptors on afferents and/or inhibitory interneurons.

## Introduction

The primary cause of death from illicit opioid use is respiratory depression caused by the activation of mu opioid receptors (MORs) in various brainstem respiratory nuclei (Dahan et al., 2001; Bateman et al., 2021). Opioid-induced respiratory depression presents with slow and irregular breathing due to inhibition in rhythmogenic and pattern-modulating respiratory nuclei (Palkovic et al., 2020; Bateman et al., 2021; Ramirez et al., 2021). This decrease in ventilation leads to decreased blood concentrations of O_2_ and increased levels of CO_2_ (Macintyre, 2001; Pattinson, 2008). Additionally, opioids also affect the hypoxic and hypercapnic chemoreflexes due to the activation of MORs, which further exaggerates opioid effects on breathing (Weil et al., 1975; Dahan et al., 2001; May et al., 2013).

Several opioid-sensitive respiratory nuclei have been implicated in the hypoxic and hypercapnic ventilatory chemoreflex responses, including the nucleus of the solitary tract (NTS) (Coates et al., 1993; Nattie and Li, 2002, 2008; Zhang et al., 2011; Zhuang et al., 2017). The NTS contains CO_2_-sensitive neurons (Dean et al., 1989; Nichols et al., 2009a) and is also the site where chemoreceptor afferents from oxygen-sensitive carotid bodies first synapse before this information is relayed from second-order neurons to upstream respiratory regions (Andresen and Kunze, 1994; Kline et al., 2010; King et al., 2012; Zoccal et al., 2014). Hypercapnia and hypoxia elicit expression of the immediate early gene, cFos, as an indicator of recent neural activity in the NTS (Jansen et al., 1996; Teppema et al., 1997; Ohtake et al., 2000; Tankersley et al., 2002; King et al., 2012).

The NTS abundantly expresses MORs (Mansour et al., 1994; Zhuang et al., 2017). Microinjection of the MOR agonist DAMGO in the caudomedial portion of the NTS inhibits both the hypercapnic and hypoxic ventilatory response in rats, which is blocked by the selective MOR antagonist CTAP (Zhang et al., 2011; Zhuang et al., 2017). Whether this inhibition is caused by somatodendritic MORs or presynaptic MORs on afferent terminals is unknown. In addition, systemic administration of morphine has been shown to induce cFos expression in the NTS, indicating possible activation of NTS neurons by opioids (Hammond et al., 1992; Grabus et al., 2004; Salas et al., 2013).

Despite significant advances in our understanding of opioid-induced respiratory depression, the mechanisms through which MOR agonism leads to impaired chemoreflexes are not well-understood. Here, we sought to assess whether NTS neurons activated during chemoreflex ventilatory responses express MORs and whether morphine would reduce hypercapnia-mediated activation. We examined the overlap between chemoreflex-sensitive neurons and opioid-sensitive neurons in the NTS by measuring cFos expression as a proxy for neuronal activation following exposure to moderate hypercapnia, hypoxia, or morphine in mice with fluorescently tagged MOR-expressing neurons. Our results imply that while a small portion of MOR-expressing neurons is activated by hypercapnia and hypoxia, the majority of chemoreflex-activated NTS neurons are not directly opioid-sensitive.

## Methods

### Animals

All experiments were approved by the Institutional Animal Care and Use Committee at the University of Florida and were in agreement with the National Institutes of Health “Guide for the Care and Use of Laboratory Animals.” Homozygous Oprm1^Cre/Cre^ mice (Liu et al., 2021) (Jackson Labs Stock #035574) were crossed with homozygous Ai9-tdTomato Cre reporter mice (Jackson Labs Stock #007909) to generate Oprm1^Cre/tdT^ mice. Oprm1^Cre/tdT^ mice (male and female, 2–6 months old) and wild-type C57BL/6J mice (male and female, 2–7 months old) were used for all experiments. Experimental groups were counterbalanced for age and sex. No apparent age or sex-dependent differences were observed, so data were pooled. All mice were bred and maintained at the University of Florida animal facility. The mice were group-housed in standard-sized plastic cages and kept on a 12-h light/dark cycle, with water and food available *ad libitum*.

### Drugs

Morphine sulfate was obtained from the National Institute on Drug Abuse Supply Program (RTI International, Research Triangle Park, NC).

### Chemoreflex and Morphine Challenges

Chemoreflex challenges were performed in two phases in separate cohorts of mice. The first phase utilized Buxco whole-body plethysmography chambers (Buxco Electronics Ltd., NT, United States). In this phase, Oprm1^Cre/tdT^ mice were acclimated to the chambers ventilated (0.5 L/min) with standard compressed room air for 1 h per day for 3 consecutive days prior to experimentation. On experiment day, mice were placed in the chambers and given a 15-min acclimation period with standard compressed air. Following this acclimation period, mice were exposed to either standard compressed air, a hypoxic challenge (10% O_2_, 90% N_2_), or a hypercapnic challenge (7% CO_2_, 21% O_2_, 72% N_2_), for 60 min. Animals were then removed from the chambers and placed in their home cage for 60 min prior to perfusion.

The second phase of chemoreflex challenges was conducted using vivoFlow whole-body plethysmography chambers (SCIREQ Inc, Montreal, QC, Canada). The mice were handled and exposed to the whole-body plethysmography system ventilated (0.5 L/min) with standard air for 1 h a day for 3 consecutive days immediately prior to experimentation. Oprm1^Cre/tdT^ and C57BL/6J wild-type mice were grouped into one of four conditions: saline injection (10 μl/g, i.p.) with standard air, saline injection with hypercapnic air (7% CO_2_, 21% O_2_, 72% N_2_), morphine injection (30 mg/kg, i.p.) with standard air, or morphine injection with hypercapnic air. On experimentation day, the mice were acclimated to the chambers for 15 min, injected with saline or morphine and exposed to standard air or hypercapnic air for 60 min. The mice were then returned to their home cages for 30 min prior to perfusion.

### Plethysmography

During the second phase of challenges, recordings of respiratory frequency and estimated tidal volume were collected using vivoFlow whole-body plethysmography and IOX2 software (SCIREQ Inc, Montreal, QC, Canada) 10–30 min post-saline or morphine (30 mg/kg) injection in mice breathing standard air or hypercapnic air. To calibrate volume changes, 10 ml of air was injected into the chambers using a 10 ml syringe prior to each recording session, in accordance with the manufacturer's instructions. Tidal volume was calculated from the integral of the inspiratory time. It is important to note that body temperature was not recorded, so tidal volume measurements are estimates. Since tidal volume is used in the calculation of minute ventilation, these measurements are also estimates. The chambers were ventilated with a constant airflow of 0.5 L/min of standard air or hypercapnic air (7% CO_2_), as described above. All plethysmography experiments were conducted at room temperature without thermoregulatory compensation. Potential breaths were rejected if the ratio of inspiratory to expiratory volume was below 70%.

### Immunohistochemistry

Following experimental exposures, the mice were deeply anesthetized using isoflurane and transcardially perfused with PBS followed by 10% formalin. Brains were removed and post-fixed overnight. Brains were then cryoprotected in 10% sucrose/PBS solution, followed by 20% sucrose/PBS solution. Free-floating coronal sections (40 μm) containing caudal NTS [-7.56 to−7.76 mm caudal to bregma (Franklin and Paxinos, 2008)] were prepared with a cryostat and stored in PBS at 4°C until staining.

The free-floating sections were washed and permeabilized with PBS-T (0.3% TritonX-100), blocked in 3% NGS in PBS-T for 1 h and incubated in primary antibody (rabbit anti-cFos [Abcam, ab190289] 1:2000 in blocking buffer) overnight. The sections were then washed in PBS-T and incubated in secondary antibody (goat anti-rabbit AlexaFluor 488 [Invitrogen, A11008] 1:500) for 2 h. The sections were washed and rinsed once in ddH_2_O before mounting with Flouromount-G DAPI (ThermoFisher) mounting medium. The sections were imaged using a confocal laser scanning microscope (Nikon A1R) with a 10X objective (N. A. 0.3).

### Image Processing and Cell Counting

Image processing was performed in FIJI (Schindelin et al., 2012) and the interactive machine learning software Ilastik (Berg et al., 2019). For each image, maximum intensity projections were generated using FIJI and imported into Ilastik. In Ilastik, a segmentation algorithm was manually trained on the set of images. Under this framework, all images analyzed with a particular algorithm receive identical treatment.

Ilastik was used to calculate features related to pixel intensity, edges, and texture for each pixel at seven different radii (0.3, 0.7, 1.0, 1.6, 3.5, 5.0, 10.0). The features calculated included: Gaussian Smoothing (intensity), Laplacian of Gaussian, Gaussian Gradient Magnitude, and Difference of Gaussians (edge detection), and Structure Tensor Eigenvalues and Hessian of Gaussian Eigenvalues (texture). The segmentation algorithm was trained on the complete set of max-intensity projections for a given region, using experimenter annotations to label a subset of pixels in each image as “cell” or “background.” A parallel random forest (VIGRA) algorithm predicts the probability that the remaining pixels are “cell” or “background” based on these annotations and the 42 calculated features.

After training, the algorithm exports a probability map for each image, representing the likelihood that each pixel constitutes part of a cell. The max-intensity projections and corresponding probability maps were then loaded into an Ilastik Object Classification Workflow, where probability thresholding and size filters were used to identify cells. Random images and the corresponding binary images were reviewed by a blinded experimenter observer to verify the accuracy of the algorithm.

The number of cFos+, tdTomato+ or co-labeled cells was determined in the caudal NTS (-7.56 to−7.76 mm caudal to bregma) for each section (2–6 sections/mouse) and averaged to determine the mean # of cFos+, tdTomato+ or co-labeled cells/section for each mouse. N values reported in Results represent the number of mice per group.

### Statistics

All statistical analyses were performed in GraphPad Prism 8. Error bars represent the standard error of the mean (SEM). Data with n > 7 were tested for normality with D'Agostino and Pearson normality test. For normally distributed data and data with *n* ≤ 7, comparisons between two groups were made using unpaired Student's two-tailed *t*-test. Comparisons between three or more groups were made using two-way ANOVA followed by Holm-Sidak *post hoc* test.

## Results

### The Hypercapnic Ventilatory Response Is Suppressed by Morphine

**Figure 1:**
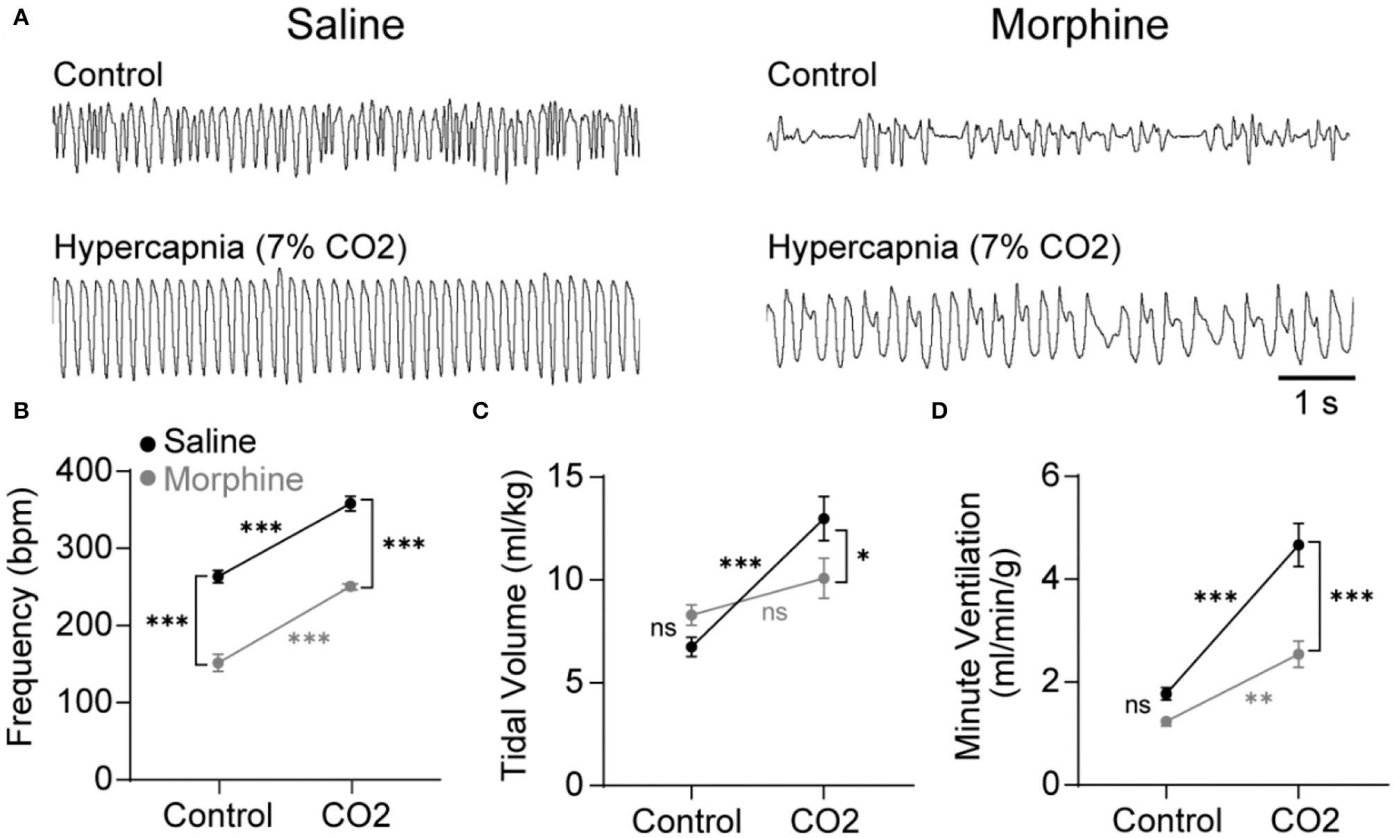
Morphine suppresses breathing during control and hypercapnic conditions in wild-type mice. Respiration was measured during control conditions (standard room air) or hypercapnia challenge (7% CO_2_) using whole-body plethysmography following an injection of saline (black symbols) or morphine (30 mg/kg; gray symbols). **(A)** Representative plethysmography traces during each of the four conditions. Scale bar applies to all traces. Inspiration is downward. **(B–D)** Saline control *n* = 11, morphine control *n* = 11, saline hypercapnia *n* = 10, morphine hypercapnia *n* = 10. Data are graphed as mean ± SEM. *p < 0.05, ***p < 0.0001, ns *p* > 0.05 by two-way ANOVA and Holm-Sidak multiple comparison's test.

Opioids attenuate the hypercapnic ventilatory response (HCVR) mediated by the NTS (Zhang et al., 2011; Zhuang et al., 2017), but whether this inhibition is caused by somatodendritic MORs or presynaptic MORs on afferent terminals is unknown. To begin to answer this question, wild-type mice were exposed to standard air or hypercapnia (7% CO2) following an injection of saline or morphine (30 mg/kg). The ventilatory effects of morphine and hypercapnia were verified using whole-body plethysmography (Figure 1). In the mice exposed to standard air (*n* = 11), morphine significantly reduced breathing frequency (saline: 264 ± 8 bpm vs. morphine: 152 ± 11 bpm, *p* < 0.0001 by two-way ANOVA and Holm-Sidak *post hoc* test, Figure 1A), but not tidal volume or minute ventilation (tidal volume = saline: 6.7 ± 0.5 ml/kg vs. morphine: 8.3 ± 0.5 ml/kg, *p* = 0.212 by two-way ANOVA and Holm-Sidak *post hoc* test, Figure 1B; minute ventilation = saline: 1.8 ± 0.1 ml/min/g vs. morphine: 1.2 ± 0.1 ml/min/g, *p* = 0.124 by two-way ANOVA and Holm-Sidak *post hoc* test, Figure 1C). Hypercapnia-induced increases in minute ventilation, breathing frequency, and tidal volume were all significantly depressed by morphine (minute ventilation in saline 4.7 ± 0.4 ml/min/g vs. morphine 2.5 ± 0.3 ml/min/g, *p* < 0.0001 by two-way ANOVA and Holm-Sidak post-test; frequency in saline 358 ± 9 bpm vs. morphine 251 ± 3 bpm, *p* < 0.0001 by two-way ANOVA and Holm-Sidak post-test; tidal volume in saline 13.0 ± 1.1 ml/kg vs. morphine 10.1 ± 1.0 ml/kg, *p* = 0.0407 by two-way ANOVA and Holm-Sidak post-test; *n* = 10 mice), consistent with established effects of morphine on the HCVR in mice and humans (Weil et al., 1975; Dahan et al., 2001).

### NTS cFos Expression Induced by Hypercapnia

**Figure 2:**
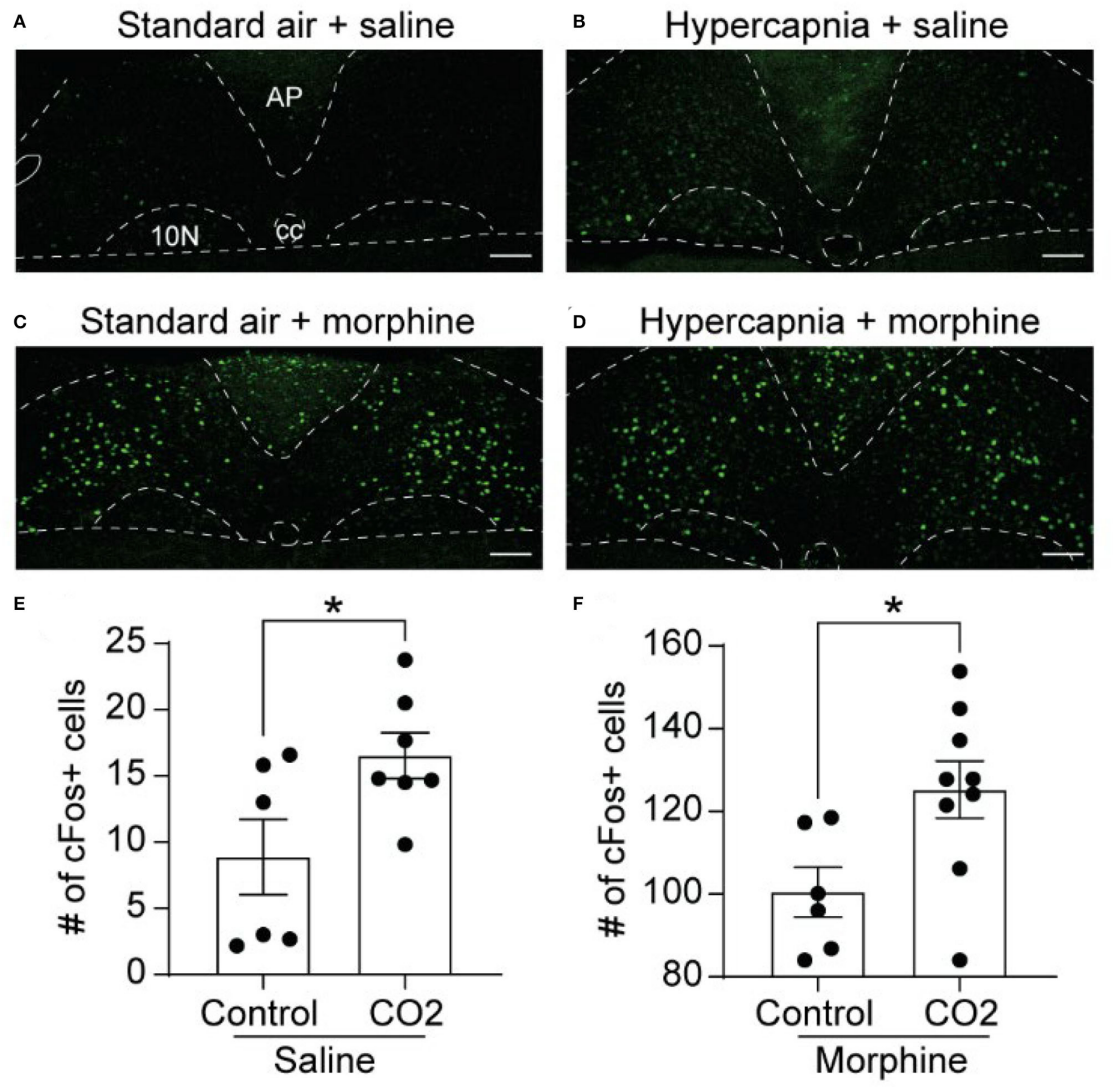
Hypercapnia and morphine activate non-overlapping populations of NTS neurons. Wild-type mice were exposed to standard room air (Control) or hypercapnia (7% CO_2_) following an injection of saline or morphine (30 mg/kg). **(A–D)** Example images of cFos immunolabeling (green) in NTS sections from mice exposed to each of the four treatment groups. Scale bar = 100 μm. AP, area postrema; cc, central canal; 10N, dorsal motor nucleus of vagus. **(E,F)** Summary data of the average number of cFos+ NTS cells per section (*n* = 6–9 mice/group, 2–6 sections/mouse). Hypercapnia increased the number of cFos+ cells in saline **(E)** and morphine **(F)** treated mice. Each data point represents the average # of cFos+ cells per section for an individual mouse. Bar and error are group mean ± SEM. *p < 0.05 by unpaired *t-*test.

We next examined the expression of cFos, as a proxy for neuronal activation, in the NTS of WT mice exposed to standard air or hypercapnia. The NTS of saline-treated, standard air-exposed mice (*n* = 6 mice, 5-6 sections/mouse) contained a low number of cFos-expressing cells, scattered throughout the region (9 ± 3 cFos+ cells/section; Figures 2A,E). The number of cFos+ cells was significantly increased in saline-treated mice that underwent a hypercapnic challenge (*n* = 7 mice, 2-6 sections/mouse: 17 ± 2 cFos+ cells/section, *p* = 0.037, unpaired *t*-test; Figures 2B,E), consistent with existing literature (Jansen et al., 1996; Teppema et al., 1997; Tankersley et al., 2002).

### NTS cFos Expression Induced by Acute Morphine

In mice exposed to standard air, morphine significantly increased the number of cFos-expressing cells in the NTS relative to saline treatment (*n* = 6 mice, 2-6 sections/mouse, 101 ± 6 cFos+ cells/section, *p* < 0.0001 by unpaired *t*-test, Figures 2C,F). These data indicate that the NTS contains a large proportion of neurons that express cFos in response to morphine administration. These neurons may or may not play a role in the HCVR. We next measured cFos expression in morphine-injected mice that were exposed to hypercapnia (*n* = 9 mice, 4–6 sections/mouse). Hypercapnia further increased the number of cFos+ cells in morphine-treated mice compared to morphine treatment in standard air (125 ± 7 cFos+ cells/section, *p* = 0.026 by unpaired *t*-test, Figures 2D,F). The results imply that hypercapnia recruits an additional population of NTS neurons that do not overlap with those activated under the influence of morphine alone.

### Hypercapnia Induces cFos Expression in MOR-Negative Cells

**Figure 3:**
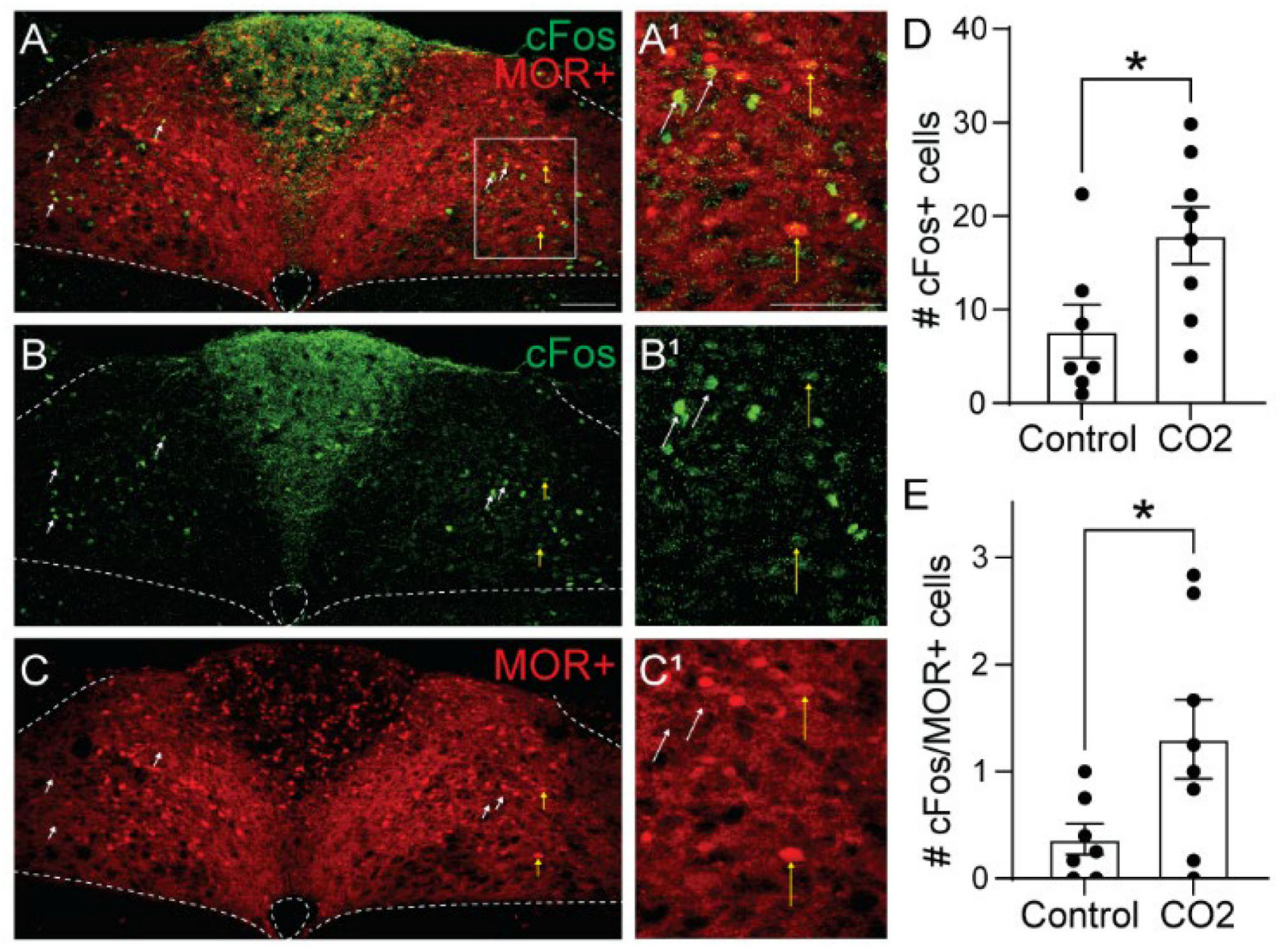
Hypercapnia activates MOR-negative neurons. Identification of cFos immunolabeling and tdTomato expression, as an indicator of MOR expression, in the NTS of Oprm1^Cre/tdT^ mice that were exposed to standard air (Control) or hypercapnic air (7% CO2). **(A–C)** Example images of cFos immunostaining (green) and MOR+ tdTomato expression (red) from a mouse exposed to hypercapnia. Very few cells co-expressed cFos and tdTomato (indicated by yellow vertical arrows). White diagonal arrows are pointing at example cells that are cFos+, but do not express MORs. **(A**^**1**^**–C**^**1**^**)** are zoomed in views of the boxed region in **(A–C)**. Scale bar = 100 μm. **(D,E)** Summary data of the average number of cFos+ NTS cells per section **(D)**, or the average number of co-labeled cFos+/MOR+ NTS cells per section **(E)** (*n* = 7–8 mice/group, 2–6 sections/mouse). Each data point represents the average # of cFos+ cells per section for an individual mouse. Bar and error are group mean ± SEM. *p < 0.05 by unpaired *t-*test.

To determine whether cells activated by hypercapnia express MORs, we crossed Oprm1^Cre/Cre^ mice with Ai9 tdTomato Cre-reporter mice to generate Ai9^tdT/+^::oprm1^Cre/+^ mice (hereby referred to as Oprm1^Cre/tdT^ mice) which express tdTomato in MOR-expressing cells. We exposed Oprm1^Cre/tdT^ mice to standard air or hypercapnia (7%) and identified cFos+, tdTomato+, and co-labeled cells in the NTS (Figure 3). There was no significant difference in the average number of tdTomato+ neurons in the NTS of hypercapnia-exposed (*n* = 8 mice, 5-6 sections/mouse) and standard air-exposed (*n* = 7 mice, 2-6 sections/mouse) mice (hypercapnia: 218 ± 39 cFos+ cells/section vs. standard air: 179 ± 20 cFos+ cells/section, *p* = 0.40 by unpaired *t*-test). In both groups, tdTomato expression in the NTS occurred in both neuronal cell bodies and neurites (Figures 3A,C), indicating MOR expression in afferents in the NTS, as well as NTS neurons themselves. Consistent with results from wild-type mice, there were significantly more cFos+ cells in the NTS of hypercapnia-exposed mice relative to standard room air-exposed mice (hypercapnia: 18 ± 3 cFos+ cells/section vs. control: 8 ± 3 cFos+ cells/section, *p* = 0.030 by unpaired *t*-test, Figure 3D). There was also a significantly higher number of cFos+/tdTomato+ co-labeled cells in the hypercapnia group relative to the control group (hypercapnia: 1.3 ± 0.4 co-labeled cells/section vs. control: 0.4 ± 0.1 co-labeled cells/section, *p*=0.044 by unpaired *t*-test, Figure 3E), indicating that some MOR-expressing NTS neurons participate in the HCVR. However, the number of cFos+/tdTomato+ co-labeled cells was very low in both groups. Only 7.6% of cFos+ cells were MOR+, and fewer than 1% of MOR+ cells were cFos+. Taken together, these data suggest that very few of the cells activated by hypercapnia are MOR+, implying that opioid inhibition of the hypercapnic ventilatory response at the level of the NTS is likely mediated by presynaptic, rather than somatodendritic inhibition.

### Hypoxia Induces cFos Expression in MOR-Negative Cells

**Figure 4:**
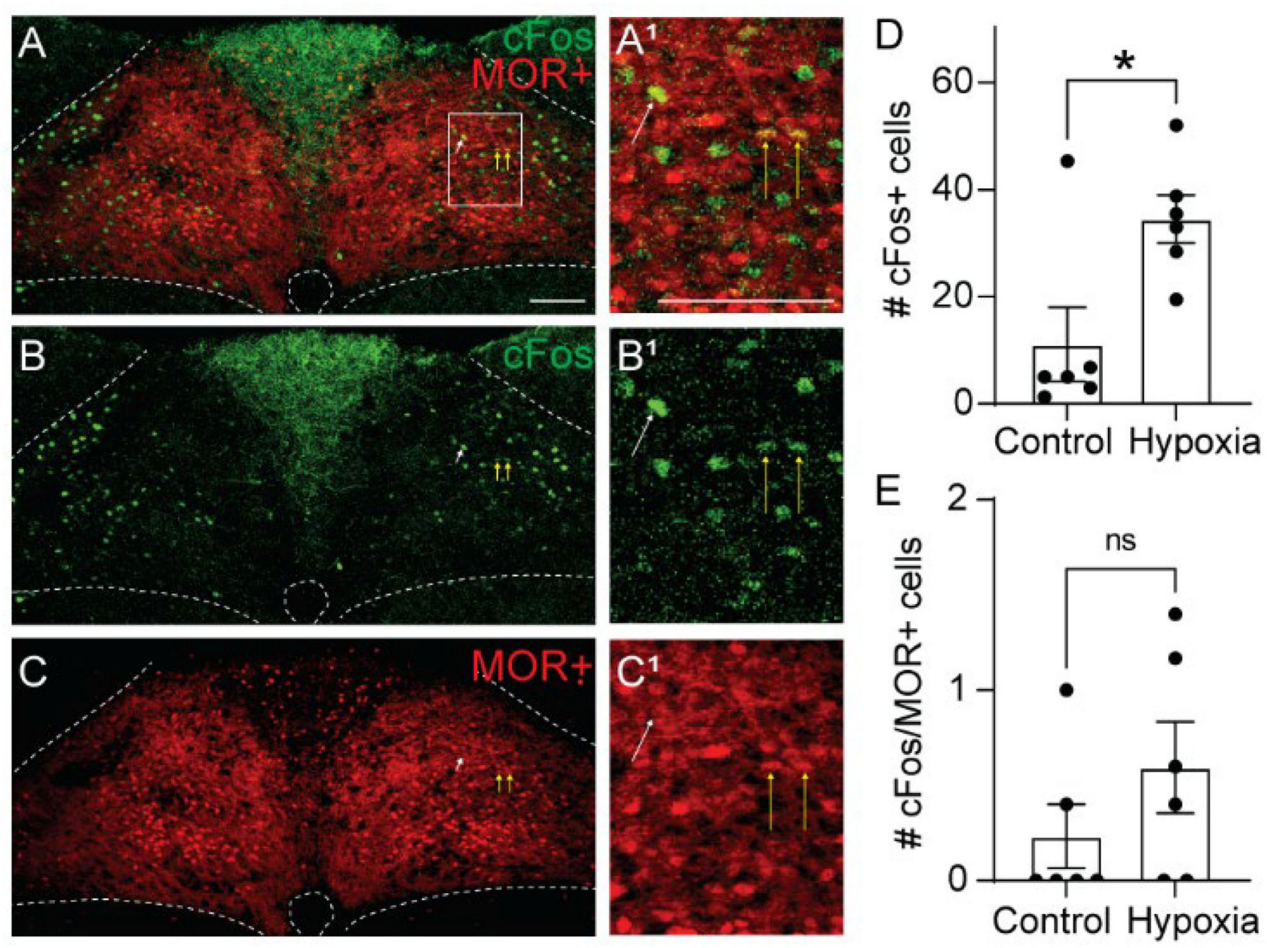
Hypoxia activates MOR-negative neurons. Identification of cFos immunolabeling and tdTomato expression, as an indicator of MOR expression, in the NTS of Oprm1^Cre/tdT^ mice that were exposed to standard air (Control) or hypoxic air (10% O2). **(A–C)** Example images of cFos immunostaining (green) and MOR+ tdTomato expression (red) from a mouse exposed to hypoxia. Very few cells co-expressed cFos and tdTomato (indicated by yellow vertical arrows). White diagonal arrows are pointing at example cells that are cFos+, but do not express MORs. **(A**^**1**^**–C**^**1**^**)** are zoomed in views of the boxed region in **(A–C)**. Scale bar = 100 μm. **(D,E)** Summary data of the average number of cFos+ NTS cells per section **(D)**, or the average number of co-labeled cFos+/MOR+ NTS cells per section **(E)** (*n* = 6 mice/group, 4–6 sections/mouse). Each data point represents the average # of cFos+ cells per section for an individual mouse. Bar and error are group mean ± SEM. *p < 0.05; ns, *p* > 0.05 by unpaired *t-*test.

Opioid-induced hypoventilation produces oxygen desaturation, in addition to the accumulation of CO2. Activation of MORs in the NTS can also significantly impair the hypoxic ventilatory response (Zhang et al., 2011; Zhuang et al., 2017). To determine whether cells activated by hypoxia express MORs, we exposed Oprm1^Cre/tdT^ mice to standard air or hypoxia (10%) and identified cFos+, tdTomato+, and co-labeled cells in the NTS (Figure 4, *n* = 6 mice/group, 4-6 sections per mouse). Consistent with prior studies (Teppema et al., 1997; Ohtake et al., 2000; King et al., 2012), there was a significantly higher number of cFos+ cells in the NTS of hypoxia-exposed mice relative to standard air-exposed mice (hypoxia: 35 ± 4 cFos+ cells/section vs. control: 11 ± 7 cFos+ cells/section, *p* = 0.017 by unpaired *t*-test, Figure 4D). However, there was no significant difference in the number of cFos+/tdTomato+ co-labeled cells between the hypoxia and control groups (hypoxia: 0.6 ± 0.2 cFos+ cells/section vs. control: 0.2 ± 0.2 cFos+ cells/section, *p* = 0.244 by unpaired *t-*test, Figure 4E). The number of co-labeled cells was very low in both standard air and hypoxia-exposed mice. Fewer than 5% of cFos+ cells were MOR+, and fewer than 1% of MOR+ cells were cFos+. Taken together, these data suggest that very few of the cells activated by hypoxia are MOR+, implying that opioid inhibition of the hypoxic ventilatory response at the level of the NTS is also likely mediated by presynaptic, rather than postsynaptic inhibition.

### Morphine Induces cFos Expression in MOR-Negative and MOR-Positive Cells

**Figure 5:**
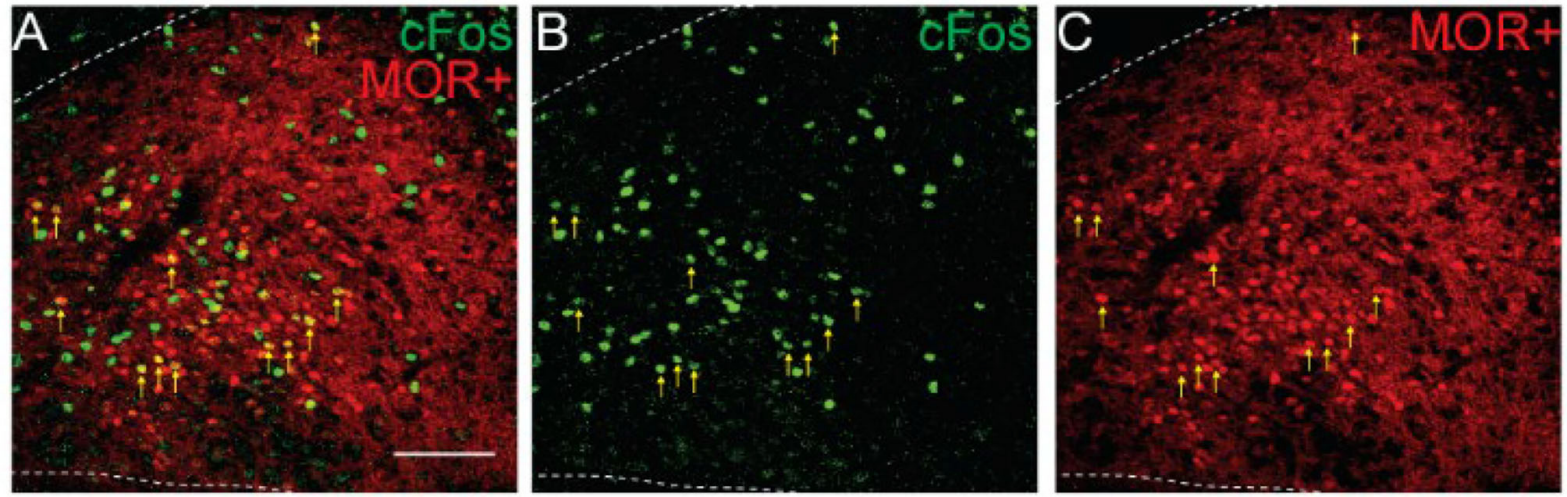
Morphine induces cFos expression in MOR-positive and MOR-negative NTS neurons. Identification of cFos immunolabeling and tdTomato expression, as an indicator of MOR expression, in the NTS of Oprm1^Cre/tdT^ mice that were injected with morphine (30 mg/kg). **(A–C)** Example images of cFos immunostaining (green) and MOR+ tdTomato expression (red). Scale bar = 100 μm. Cells that co-expressed cFos and tdTomato are indicated by yellow vertical arrows.

Since activation of MORs by morphine could directly lead to cFos expression independent of neuronal activation (Shoda et al., 2001), we next tested if NTS cells activated by morphine treatment expressed MORs (*n* = 2 mice, 3-4 sections/mouse). Only 6.2% of tdTomato+ cells was cFos+, while 15.1% of the cFos+ cells was tdTomato+ indicating they expressed MORs (Figure 5, 299 ± 92 tdTomato+ cells/section; 101 ± 37 cFos+ cells/section; 15 ± 5 co-labeled cFos+/MOR+ cells/section). This finding that morphine administration induces cFos expression in both MOR-positive and MOR-negative cells, suggests at least two potential pathways by which morphine can induce cFos expression in NTS cells. While some cells may express cFos due to direct signaling pathways from co-expressed postsynaptic receptors, the vast majority of cFos expression induced in the NTS by morphine is indirect, and likely due to neuronal activation.

Since Oprm1^Cre/tdT^ mice lose a functional copy of MOR (Liu et al., 2021), it was important to determine if morphine is still effective in these mice. In Oprm1^Cre/tdT^ mice, morphine (30 mg/kg) did cause respiratory depression compared to saline-treated controls (*n* = 4/group). Morphine reduced minute ventilation (saline-treated: 2.1 ± 0.1 ml/min/g vs. morphine-treated: 1.0 ± 0.1 ml/min/g, *p* = 0.003 by unpaired *t-*test) due to a significant reduction in breathing frequency (saline-treated: 280 ± 9 bpm vs. morphine-treated: 172 ±7 bpm, *p* < 0.0001 by unpaired *t-*test) and tidal volume (saline-treated: 7.6 ± 0.5 ml/g vs. morphine-treated: 5.7 ± 0.5 ml/g, *p* = 0.029 by unpaired *t-*test). Importantly, baseline respiration in Oprm1^Cre/tdT^ mice was similar to wild-type mice (minute ventilation *p* = 0.121, frequency *p* = 0.271, tidal volume *p* = 0.327, unpaired *t-*tests). Morphine-induced respiratory depression in Oprm1^Cre/tdT^ mice also manifests similarly to wild-type mice (minute ventilation *p* = 0.162, frequency *p* = 0.306, unpaired *t-*tests).

## Discussion

While our knowledge about the mechanisms and cellular basis of opioid-induced respiratory depression has significantly increased in recent years (Bateman et al., 2021; Ramirez et al., 2021), the mechanisms through which opioids lead to impaired chemoreflexes are not well-understood. The goal of this study was to determine the amount of overlap between chemoreflex-sensitive neurons and opioid-sensitive neurons in the NTS by measuring cFos expression as a proxy for neuronal activation in mice with fluorescently tagged MOR-expressing neurons. We hypothesized that hypercapnia would activate MOR-expressing neurons, and that morphine would reduce this hypercapnia-mediated activation. On the contrary, our results indicate that although MORs are expressed in neurons and neurites in the NTS, most neurons that are activated by hypercapnia do not express MORs. Similarly, most NTS neurons that are activated by hypoxia also do not express MORs. Thus, opioid effects on hypercapnic and hypoxic ventilatory responses in the NTS (Zhang et al., 2011; Zhuang et al., 2017) are indirectly mediated.

### Morphine Activation of NTS Neurons

Opioid receptors are inhibitory G protein-coupled receptors that inhibit neuronal activity through hyperpolarization and inhibition of neurotransmitter release. Despite this, morphine significantly increased cFos expression in the NTS, consistent with previous studies (Hammond et al., 1992; Grabus et al., 2004; Salas et al., 2013). There are multiple mechanisms by which this could occur. First, opioids can excite neurons by disinhibition (i.e., inhibition of tonic GABA release) (Johnson and North, 1992; Lau et al., 2020). The NTS contains numerous GABAergic interneurons and receives GABAergic afferent projections from other areas (Fong et al., 2005; Bailey et al., 2008). The mu opioid agonist endomorphin-1 inhibits spontaneous GABAergic neurotransmission in the NTS and hyperpolarizes a portion of GABAergic NTS interneurons (Glatzer et al., 2007). In addition, endomorphin-1 inhibits solitary tract stimulation-evoked glutamate release onto GABAergic neurons in the NTS (Glatzer et al., 2007), which could also decrease GABAergic interneuron activity. Thus, disinhibition is a likely mechanism by which morphine increased cFos expression in the NTS and could lead to impairments in hypoxic ventilatory responses (Tabata et al., 2001; Chung et al., 2006). Second, MOR-coupled intracellular signaling cascades can lead to the induction of cFos expression in the absence of neuronal activation (Shoda et al., 2001). This signaling mechanism would only induce cFos expression in neurons that express MORs. Since only a small percentage of cFos expressing cells co-expressed MORs, this is likely a minor mechanism by which morphine-induced cFos expression in the NTS. Finally, since morphine reduces ventilation, which can lead to hypoxemia and accumulation of CO_2_, morphine could have activated neurons through chemoreflex pathways. There were more cFos expressing neurons in the NTS from mice that received morphine and a hypercapnic challenge. Morphine exacerbation of hypercapnia may recruit additional non-opioid-sensitive CO_2_-sensitive NTS neurons. Since morphine also induces hypoxemia, the recruitment of additional hypoxia-sensitive neurons is also possible. Interestingly, withdrawal from chronic morphine treatment also induces cFos expression in the NTS (Stornetta et al., 1993; Laorden et al., 2002; Mannelli et al., 2004; Benavides et al., 2005). Presumably, the neurons activated by acute morphine and morphine withdrawal should be distinct populations, but this remains to be determined.

### Presynaptic MORs in the NTS

Our findings that neurons activated by hypercapnia and hypoxia do not express MORs suggest that the effects of opioid agonist in the NTS (Zhang et al., 2011; Zhuang et al., 2017) are indirect and possibly mediated by presynaptic MORs on axon terminals. The NTS contained a significant amount of MOR-expressing neurons and neurites compared to the surrounding area, consistent with previous reports (Mansour et al., 1994; Aicher et al., 2000; Zhuang et al., 2017), and a substantial amount of MOR expression in the NTS is in afferents (Aicher et al., 2000). The NTS is the first relay for several cardiorespiratory afferents, including lung and airway vagal afferents and carotid body chemoreceptor afferents (Kubin et al., 2006). Ultrastructural microscopy identified MORs on vagal afferent terminals in the medial NTS, which primarily synapsed onto non-MOR-expressing NTS neurons, suggesting that MORs modulate NTS neurons either presynaptically or postsynaptically, but not both (Aicher et al., 2000). The mu opioid agonist endomorphin-1 inhibits solitary tract stimulation-evoked glutamate release onto GABAergic neurons in the NTS, supporting the functional expression of MORs on solitary tract axon terminals (Glatzer et al., 2007). Presynaptic MORs also inhibit GABAergic and glutamatergic neurotransmission in the NTS (Rhim et al., 1993; Glatzer et al., 2007). MOR agonist injection into the NTS also inhibited bronchopulmonary C-fiber-induced reflexes in the NTS (Zhuang et al., 2017). The relative contribution of these afferent-specific presynaptic MORs in the hypercapnic and hypoxic ventilatory response remains to be determined.

### Other Brain Areas Involved in Opioid Suppression of Chemoreflexes

Opioid suppression of hypercapnic and hypoxic ventilatory responses could be due to activation of MORs in other chemosensitive areas outside the NTS as well. Injection of opioid into the caudal medullary raphe suppresses the hypoxic and hypercapnic ventilatory responses in anesthetized rats (Zhang et al., 2007, 2009). In addition, locus coeruleus neurons are involved in the hypercapnic ventilatory response (Biancardi et al., 2008; Oliveira et al., 2017; Magalhães et al., 2018) and are inhibited by MORs (North and Williams, 1985; Levitt and Williams, 2012). One area that is unlikely to mediate opioid impairment of chemoreflexes is the carotid bodies. Although MORs are expressed in the carotid bodies, transection of the carotid sinus nerve enhanced (rather than reduced) morphine-induced suppression of the hypoxic and hypercapnic ventilatory response (Baby et al., 2018).

### NTS Endogenous Opioids in Physiological Responses

Ventilation is enhanced in mice lacking mu opioid receptors (Dahan et al., 2001), suggesting endogenous opioids influence the control of breathing. The NTS is a potential source of endogenous opioids. The endogenous opioid endomorphins are abundantly expressed in the caudal NTS (Pierce and Wessendorf, 2000; Greenwell et al., 2007), and endomorphin-2 containing axon terminals oppose dendritic MORs in the NTS (Silverman et al., 2005). Selective stimulation of proopiomelanocortin (POMC) neurons in the NTS suppresses breathing, which is blocked by the opioid antagonist naloxone (Cerritelli et al., 2016), suggesting the release of opioid peptide from these neurons could modulate breathing. Furthermore, vagal afferents into the NTS contain endomorphin-2 (Silverman et al., 2005), and stimulation of the NTS or the vagus nerve is analgesic (Lewis et al., 1987; Kirchner et al., 2006) implicating the NTS as an endogenous integrator of both pain and breathing (Boscan et al., 2002). Presumably, endogenous opioids in the NTS could also modulate hypoxic and hypercapnic responses, and perhaps adaptations that occur in these responses during chronic hypoxia (Chung et al., 2006; Powell, 2007; Nichols et al., 2009b).

## Conclusions

Here, we identified that NTS neurons activated by hypercapnia and hypoxia do not express MORs, ruling out the direct effects of opioids on these neurons. More likely presynaptic MORs on axon terminals and/or MORs on inhibitory interneurons predominantly mediate opioid suppression of chemoreflexes in the NTS. The specific afferents and synaptic target suppressed by MORs remain to be elucidated. Furthermore, the role of endogenous opioids and adaptations that could occur in these afferent-specific synapses during chronic opioid or altered chemoreception states are unexplored future directions.

## Data Availability Statement

The raw data supporting the conclusions of this article will be made available by the authors, without undue reservation.

## Ethics Statement

The animal study was reviewed and approved by Institutional Animal Care and Use Committee at the University of Florida.

## Author Contributions

EL and AV designed research. SM and BR performed experiments and analyzed data. SM, AV, and EL prepared figures and drafted the manuscript. All authors revised and approved the final version of the manuscript.

## Funding

This work was supported by the National Institutes of Health Grants R01DA047978 and OT2OD023854 to EL and K99HL159232 to AV.

## Conflict of Interest

The authors declare that the research was conducted in the absence of any commercial or financial relationships that could be construed as a potential conflict of interest.

## Publisher's Note

All claims expressed in this article are solely those of the authors and do not necessarily represent those of their affiliated organizations, or those of the publisher, the editors and the reviewers. Any product that may be evaluated in this article, or claim that may be made by its manufacturer, is not guaranteed or endorsed by the publisher.

## References

[B1] Aicher S. A. Goldberg A. Sharma S. Pickel V. M. (2000). μ-opioid receptors are present in vagal afferents and their dendritic targets in the medial nucleus tractus solitarius. J. Comp. Neurol. 422, 181–190. 10.1002/(SICI)1096-9861(20000626)422:2<181::AID-CNE3>3.0.CO;2-G 10842226

[B2] Andresen M. C. Kunze D. L. (1994). Nucleus tractus solitarius—gateway to neural circulatory control. Annu. Rev. Physiol. 56, 93–116. 10.1146/annurev.ph.56.030194.000521 7912060

[B3] Baby S. M. Gruber R. B. Young A. P. MacFarlane P. M. Teppema L. J. Lewis S. J. (2018). Bilateral carotid sinus nerve transection exacerbates morphine-induced respiratory depression. Eur. J. Pharmacol. 834, 17–29. 10.1016/j.ejphar.2018.07.018 30012498 PMC6091892

[B4] Bailey T. W. Appleyard S. M. Jin Y.-H. Andresen M. C. (2008). Organization and properties of GABAergic neurons in solitary tract nucleus (NTS). J. Neurophysiol. 99, 1712–1722. 10.1152/jn.00038.2008 18272881

[B5] Bateman J. T. Saunders S. E. Levitt E. S. (2021). Understanding and countering opioid-induced respiratory depression. Br. J Pharmacol. 10.1111/bph.15580. [Epub ahead of print]. PMC8997313 34089181

[B6] Benavides M. Laorden M. L. Marín M. T. Milanés M. V. (2005). Role of PKC-α,γ isoforms in regulation of c-Fos and TH expression after naloxone-induced morphine withdrawal in the hypothalamic PVN and medulla oblongata catecholaminergic cell groups. J. Neurochem. 95, 1249–1258. 10.1111/j.1471-4159.2005.03445.x 16190878

[B7] Berg S. Kutra D. Kroeger T. Straehle C. N. Kausler B. X. Haubold C.. (2019). ilastik: interactive machine learning for (bio)image analysis. Nat. Methods 16, 1226–1232. 10.1038/s41592-019-0582-9 31570887

[B8] Biancardi V. Bícego K. C. Almeida M. C. Gargaglioni L. H. (2008). Locus coeruleus noradrenergic neurons and CO2 drive to breathing. Pflügers Arch. Eur. J. Physiol. 455, 1119–1128. 10.1007/s00424-007-0338-8 17851683

[B9] Boscan P. Pickering A. E. Paton J. F. R. (2002). The nucleus of the solitary tract: an integrating station for nociceptive and cardiorespiratory afferents. Exp. Physiol. 87, 259–266. 10.1113/eph8702353 11856972

[B10] Cerritelli S. Hirschberg S. Hill R. Balthasar N. Pickering A. E. (2016). Activation of brainstem pro-opiomelanocortin neurons produces opioidergic analgesia, bradycardia and bradypnoea. PLoS ONE 11, e0153187. 10.1371/journal.pone.0153187 27077912 PMC4831707

[B11] Chung S. Ivy G. O. Reid S. G. (2006). GABA-mediated neurotransmission in the nucleus of the solitary tract alters resting ventilation following exposure to chronic hypoxia in conscious rats. Am. J. Physiol. Regulatory Integr. Comp. Physiol. 291, R1449–R1456. 10.1152/ajpregu.00645.2005 16778062

[B12] Coates E. L. Li A. Nattie E. E. (1993). Widespread sites of brain stem ventilatory chemoreceptors. J. Appl. Physiol. 75, 5–14. 10.1152/jappl.1993.75.1.5 8376301

[B13] Dahan A. Sarton E. Teppema L. Olievier C. Nieuwenhuijs D. Matthes H. W.. (2001). Anesthetic potency and influence of morphine and sevoflurane on respiration in mu-opioid receptor knockout mice. Anesthesiology 94, 824–832. 10.1097/00000542-200105000-00021 11388534

[B14] Dean J. B. Lawing W. L. Millhorn D. E. (1989). CO2 decreases membrane conductance and depolarizes neurons in the nucleus tractus solitarii. Exp. Brain Res. 76, 656–661. 10.1007/BF00248922 2507342

[B15] Fong A. Y. Stornetta R. L. Foley C. M. Potts J. T. (2005). Immunohistochemical localization of GAD67-expressing neurons and processes in the rat brainstem: Subregional distribution in the nucleus tractus solitarius. J. Comp. Neurol. 493, 274–290. 10.1002/cne.20758 16255028

[B16] Franklin K. Paxinos G. (2008). The Mouse Brain in Stereotaxic Coordinates, Compact - 3rd Edition. Academic Press.

[B17] Glatzer N. R. Derbenev A. V. Banfield B. W. Smith B. N. (2007). Endomorphin-1 modulates intrinsic inhibition in the dorsal vagal complex. J. Neurophysiol. 98, 1591–1599. 10.1152/jn.00336.2007 17615134

[B18] Grabus S. D. Glowa J. R. Riley A. L. (2004). Morphine- and cocaine-induced c-Fos levels in Lewis and Fischer rat strains. Brain Res. 998, 20–28. 10.1016/j.brainres.2003.11.007 14725964

[B19] Greenwell T. N. Martin-Schild S. Inglis F. M. Zadina J. E. (2007). Colocalization and shared distribution of endomorphins with substance P, calcitonin gene-related peptide, γ-aminobutyric acid, and the mu opioid receptor. J. Comp. Neurol. 503, 319–333. 10.1002/cne.21374 17492626

[B20] Hammond D. L. Presley R. Gogas K. R. Basbaum A. I. (1992). Morphine or U-50,488 suppresses fos protein-like immunoreactivity in the spinal cord and nucleus tractus solitarii evoked by a noxious visceral stimulus in the rat. J. Comp. Neurol. 315, 244–253. 10.1002/cne.903150210 1545011

[B21] Jansen A. H. Nance D. M. Liu P. Weisman H. Chernick V. (1996). Effect of sinus denervation and vagotomy on c-fos expression in the nucleus tractus solitarius after exposure to C02. Pflügers Arch. 431, 876–881. 10.1007/s004240050080 8927504

[B22] Johnson S. W. North R. A. (1992). Opioids excite dopamine neurons by hyperpolarization of local interneurons. J. Neurosci. 12, 483–488. 10.1523/JNEUROSCI.12-02-00483.1992 1346804 PMC6575608

[B23] King T. L. Heesch C. M. Clark C. G. Kline D. D. Hasser E. M. (2012). Hypoxia activates nucleus tractus solitarii neurons projecting to the paraventricular nucleus of the hypothalamus. Am. J. Physiol. 302, R1219–R1232. 10.1152/ajpregu.00028.2012 22403798 PMC3362152

[B24] Kirchner A. Stefan H. Bastian K. Birklein F. (2006). Vagus nerve stimulation suppresses pain but has limited effects on neurogenic inflammation in humans. Eur. J. Pain 10, 449–449. 10.1016/j.ejpain.2005.06.005 16125425

[B25] Kline D. D. King T. L. Austgen J. R. Heesch C. M. Hasser E. M. (2010). Sensory afferent and hypoxia-mediated activation of nucleus tractus solitarius neurons that project to the rostral ventrolateral medulla. Neuroscience 167, 510–527. 10.1016/j.neuroscience.2010.02.012 20153814 PMC2849863

[B26] Kubin L. Alheid G. F. Zuperku E. J. McCrimmon D. R. (2006). Central pathways of pulmonary and lower airway vagal afferents. J. Appl. Physiol. 101, 618–627. 10.1152/japplphysiol.00252.2006 16645192 PMC4503231

[B27] Laorden M. L. Castells M. T. Milanés M. V. (2002). Effects of morphine and morphine withdrawal on brainstem neurons innervating hypothalamic nuclei that control thepituitary-adrenocortical axis in rats. Br. J. Pharmacol. 136, 67–75. 10.1038/sj.bjp.0704684 11976269 PMC1762112

[B28] Lau B. K. Winters B. L. Vaughan C. W. (2020). Opioid presynaptic disinhibition of the midbrain periaqueductal grey descending analgesic pathway. Br. J. Pharmacol. 177, 2320–2332. 10.1111/bph.14982 31971607 PMC7174888

[B29] Levitt E. S. Williams J. T. (2012). Morphine desensitization and cellular tolerance are distinguished in rat locus ceruleus neurons. Mol. Pharmacol. 82, 983–992. 10.1124/mol.112.081547 22914548 PMC3477235

[B30] Lewis J. W. Baldrighi G. Akil H. (1987). A possible interface between autonomic function and pain control: opioid analgesia and the nucleus tractus solitarius. Brain Res. 424, 65–70. 10.1016/0006-8993(87)91193-0 3319042

[B31] Liu S. Kim D.-I. Oh T. G. Pao G. M. Kim J.-H. Palmiter R. D.. (2021). Neural basis of opioid-induced respiratory depression and its rescue. Proc. Natl. Acad. Sci. U.S.A. 118, e2022134118. 10.1073/pnas.2022134118 34074761 PMC8201770

[B32] Macintyre P. E. (2001). Safety and efficacy of patient-controlled analgesia. Br. J. Anaesthesia 87, 36–46. 10.1093/bja/87.1.36 11460812

[B33] Magalhães K. S. Spiller P. F. Silva M. P. da Kuntze L. B. Paton J. F. R. Machado B. H.. (2018). Locus Coeruleus as a vigilance centre for active inspiration and expiration in rats. Sci. Rep-uk 8:15654. 10.1038/s41598-018-34047-w 30353035 PMC6199338

[B34] Mannelli P. Gottheil E. Peoples J. F. Oropeza V. C. Bockstaele E. J. V. (2004). Chronic very low dose naltrexone administration attenuates opioid withdrawal expression. Biol. Psychiat. 56, 261–268. 10.1016/j.biopsych.2004.05.013 15312814

[B35] Mansour A. Fox C. A. Burke S. Meng F. Thompson R. C. Akil H.. (1994). Mu, delta, and kappa opioid receptor mRNA expression in the rat CNS: An in situ hybridization study. J. Comp. Neurol. 350, 412–438. 10.1002/cne.903500307 7884049

[B36] May W. J. Gruber R. B. Discala J. F. Puskovic V. Henderson F. Palmer L. A.. (2013). Morphine has latent deleterious effects on the ventilatory responses to a hypoxic challenge^*^. Open J. Mol. Integr. Physiol. 2013, 166–180. 10.4236/ojmip.2013.34022 25045593 PMC4103751

[B37] Nattie E. Li A. (2008). Muscimol dialysis into the caudal aspect of the Nucleus tractus solitarii of conscious rats inhibits chemoreception. Resp. Physiol. Neurobi. 164, 394–400. 10.1016/j.resp.2008.09.004 18824146 PMC4793890

[B38] Nattie E. E. Li A. (2002). CO2 dialysis in nucleus tractus solitarius region of rat increases ventilation in sleep and wakefulness. J. Appl. Physiol. 92, 2119–2130. 10.1152/japplphysiol.01128.2001 11960965

[B39] Nichols N. L. Mulkey D. K. Wilkinson K. A. Powell F. L. Dean J. B. Putnam R. W. (2009a). Characterization of the chemosensitive response of individual solitary complex neurons from adult rats. Am. J. Physiol. 296, R763–R773. 10.1152/ajpregu.90769.2008 19144749 PMC2666395

[B40] Nichols N. L. Wilkinson K. A. Powell F. L. Dean J. B. Putnam R. W. (2009b). Chronic hypoxia suppresses the CO2 response of solitary complex (SC) neurons from rats. Resp. Physiol. Neurobi. 168, 272–280. 10.1016/j.resp.2009.07.012 19619674 PMC2750817

[B41] North R. A. Williams J. T. (1985). On the potassium conductance increased by opioids in rat locus coeruleus neurones. J. Physiol. 364, 265–280. 10.1113/jphysiol.1985.sp015743 2411916 PMC1192968

[B42] Ohtake P. J. Simakajornboon N. Fehniger M. D. Xue Y.-D. Gozal D. (2000). N-Methyl-d-aspartate receptor expression in the nucleus tractus solitarii and maturation of hypoxic ventilatory response in the rat. Am. J. Resp. Crit. Care 162, 1140–1147. 10.1164/ajrccm.162.3.9903094 10988143

[B43] Oliveira L. M. Tuppy M. Moreira T. S. Takakura A. C. (2017). Role of the locus coeruleus catecholaminergic neurons in the chemosensory control of breathing in a Parkinson's disease model. Exp. Neurol. 293, 172–180. 10.1016/j.expneurol.2017.04.006 28431876

[B44] Palkovic B. Marchenko V. Zuperku E. J. Stuth E. A. E. Stucke A. G. (2020). Multi-level regulation of opioid-induced respiratory depression. Physiology 35, 391–404. 10.1152/physiol.00015.2020 33052772 PMC7864237

[B45] Pattinson K. T. S. (2008). Opioids and the control of respiration. Br. J. Anaesthesia 100, 747–758. 10.1093/bja/aen094 18456641

[B46] Pierce T. L. Wessendorf M. W. (2000). Immunocytochemical mapping of endomorphin-2-immunoreactivity in rat brain. J. Chem. Neuroanat. 18, 181–207. 10.1016/S0891-0618(00)00042-9 10781736

[B47] Powell F. L. (2007). The influence of chronic hypoxia upon chemoreception. Resp. Physiol. Neurobi. 157, 154–161. 10.1016/j.resp.2007.01.009 17291837 PMC1964780

[B48] Ramirez J.-M. Burgraff N. J. Wei A. D. Baertsch N. A. Varga A. G. Baghdoyan H. A.. (2021). Neuronal mechanisms underlying opioid-induced respiratory depression: our current understanding. J. Neurophysiol. 125, 1899–1919. 10.1152/jn.00017.2021 33826874 PMC8424565

[B49] Rhim H. Glaum S. Miller R. (1993). Selective opioid agonists modulate afferent transmission in the rat nucleus tractus solitarius. J. Pharmacol. Exp. Ther. 264, 795–800. 8094753

[B50] Salas E. Alonso E. Polanco M. J. Cano M. V. Ruiz-Gayo M. Alguacil L. F. (2013). Differential regulation of CDK5 and c-Fos expression by morphine in the brain of Lewis and Fischer 344 rat strains. Neuroscience 230, 151–156. 10.1016/j.neuroscience.2012.11.001 23153991

[B51] Schindelin J. Arganda-Carreras I. Frise E. Kaynig V. Longair M. Pietzsch T.. (2012). Fiji: an open-source platform for biological-image analysis. Nat. Methods 9, 676–682. 10.1038/nmeth.2019 22743772 PMC3855844

[B52] Shoda T. Fukuda K. Uga H. Mima H. Morikawa H. (2001). Activation of μ-opioid receptor induces expression of c-fos and junbvia mitogen-activated protein kinase cascade. Anesthesiology 95, 983–989. 10.1097/00000542-200110000-00030 11605942

[B53] Silverman M. B. Hermes S. M. Zadina J. E. Aicher S. A. (2005). Mu-opioid receptor is present in dendritic targets of Endomorphin-2 axon terminals in the nuclei of the solitary tract. Neuroscience 135, 887–896. 10.1016/j.neuroscience.2005.06.072 16154285

[B54] Stornetta R. L. Norton F. E. Guyenet P. G. (1993). Autonomic areas of rat brain exhibit increased Fos-like immunoreactivity during opiate withdrawal in rats. Brain Res. 624, 19–28. 10.1016/0006-8993(93)90055-R 7902768

[B55] Tabata M. Kurosawa H. Kikuchi Y. Hida W. Ogawa H. Okabe S.. (2001). Role of GABA within the nucleus tractus solitarii in the hypoxic ventilatory decline of awake rats. Am. J. Physiol. 281, R1411–R1419. 10.1152/ajpregu.2001.281.5.R1411 11641110

[B56] Tankersley C. G. Haxhiu M. A. Gauda E. B. (2002). Differential CO2-induced c-fos gene expression in the nucleus tractus solitarii of inbred mouse strains. J. Appl. Physiol. 92, 1277–1284. 10.1152/japplphysiol.00609.2001 11842068

[B57] Teppema L. J. Veening J. G. Kranenburg A. Dahan A. Berkenbosch A. Olievier C. (1997). Expression of c-fos in the rat brainstem after exposure to hypoxia and to normoxic and hyperoxic hypercapnia. J. Comp. Neurol. 388, 169–190. 9368836 10.1002/(sici)1096-9861(19971117)388:2<169::aid-cne1>3.0.co;2-#

[B58] Weil J. V. McCullough R. E. Kline J. S. Sodal I. E. (1975). Diminished ventilatory response to hypoxia and hypercapnia after morphine in normal man. New Engl. J. Med. 292, 1103–1106. 10.1056/NEJM197505222922106 1128555

[B59] Zhang Z. Xu F. Zhang C. Liang X. (2007). Activation of opioid mu receptors in caudal medullary raphe region inhibits the ventilatory response to hypercapnia in anesthetized rats. Anesthesiology 107, 288–297. 10.1097/01.anes.0000270760.46821.67 17667574

[B60] Zhang Z. Xu F. Zhang C. Liang X. (2009). Opioid μ-receptors in medullary raphe region affect the hypoxic ventilation in anesthetized rats. Resp. Physiol. Neurobi. 168, 281–288. 10.1016/j.resp.2009.07.015 19632358 PMC3438222

[B61] Zhang Z. Zhuang J. Zhang C. Xu F. (2011). Activation of opioid $mu$-receptors in the commissural subdivision of the nucleus tractus solitarius abolishes the ventilatory response to hypoxia in anesthetized rats. Anesthesiology 115, 353–363. 10.1097/ALN.0b013e318224cc1f 21716092

[B62] Zhuang J. Gao X. Gao F. Xu F. (2017). Mu-opioid receptors in the caudomedial NTS are critical for respiratory responses to stimulation of bronchopulmonary C-fibers and carotid body in conscious rats. Resp. Physiol. Neurobi. 235, 71–78. 10.1016/j.resp.2016.10.004 27743812

[B63] Zoccal D. B. Furuya W. I. Bassi M. Colombari D. S. A. Colombari E. (2014). The nucleus of the solitary tract and the coordination of respiratory and sympathetic activities. Front. Physiol. 5, 238. 10.3389/fphys.2014.00238 25009507 PMC4070480

